# Chemotactic Cues for NOTCH1-Dependent Leukemia

**DOI:** 10.3389/fimmu.2018.00633

**Published:** 2018-04-03

**Authors:** Erich Piovan, Valeria Tosello, Alberto Amadori, Paola Zanovello

**Affiliations:** ^1^UOC Immunologia e Diagnostica Molecolare Oncologica, Istituto Oncologico Veneto IOV—IRCCS, Padova, Italy; ^2^Dipartimento di Scienze Chirurgiche, Oncologiche e Gastroenterologiche, Università di Padova, Padova, Italy

**Keywords:** T-cell acute lymphoblastic leukemia, chemokines, CXC-chemokine receptor 4, stromal-derived factor-1, NOTCH, CXCR7, infiltration

## Abstract

The NOTCH signaling pathway is a conserved signaling cascade that regulates many aspects of development and homeostasis in multiple organ systems. Aberrant activity of this signaling pathway is linked to the initiation and progression of several hematological malignancies, exemplified by T-cell acute lymphoblastic leukemia (T-ALL). Interestingly, frequent non-mutational activation of NOTCH1 signaling has recently been demonstrated in B-cell chronic lymphocytic leukemia (B-CLL), significantly extending the pathogenic significance of this pathway in B-CLL. Leukemia patients often present with high-blood cell counts, diffuse disease with infiltration of the bone marrow, secondary lymphoid organs, and diffusion to the central nervous system (CNS). Chemokines are chemotactic cytokines that regulate migration of cells between tissues and the positioning and interactions of cells within tissue. Homeostatic chemokines and their receptors have been implicated in regulating organ-specific infiltration, but may also directly and indirectly modulate tumor growth. Recently, oncogenic NOTCH1 has been shown to regulate infiltration of leukemic cells into the CNS hijacking the CC-chemokine ligand 19/CC-chemokine receptor 7 chemokine axis. In addition, a crucial role for the homing receptor axis CXC-chemokine ligand 12/CXC-chemokine receptor 4 has been demonstrated in leukemia maintenance and progression. Moreover, the CCL25/CCR9 axis has been implicated in the homing of leukemic cells into the gut, particularly in the presence of phosphatase and tensin homolog tumor suppressor loss. In this review, we summarize the latest developments regarding the role of NOTCH signaling in regulating the chemotactic microenvironmental cues involved in the generation and progression of T-ALL and compare these findings to B-CLL.

## Introduction

The NOTCH signaling cascade is an evolutionarily conserved signaling pathway that in mammals consists of a family of four transmembrane receptors (NOTCH1, NOTCH2, NOTCH3, and NOTCH4) (1) and five ligands of the Delta-Serrate-Lag family [jagged 1 (JAG1), JAG2, delta-like 1 (DLL1), DLL3 and DLL4] (2). This signaling system plays a crucial role in regulating development and tissue homeostasis (3). Given the important role played by NOTCH signaling in regulating key cellular traits such as differentiation, proliferation, and apoptosis, it is perhaps not surprising that deregulation of NOTCH has been implicated in the pathogenesis of a variety of malignancies (4, 5). In this regard, the most firmly established evidence for altered NOTCH signaling in cancer is represented by activating *NOTCH1* receptor mutations found in over 50–60% of T-cell acute lymphoblastic leukemia (T-ALL) cases (6). In addition, 8–30% of T-ALLs harbor mutations in F-box and WD repeat domain containing 7 (*FBXW7*), a protein that normally promotes NOTCH1 ubiquitination and degradation, which lead to increased NOTCH1 protein stability (7, 8). Moreover, paracrine mechanisms that result in NOTCH1 or NOTCH3 signaling upregulation or rare mutations in *NOTCH3* (9) could contribute to T-ALL. Further, aberrant expression of the NOTCH ligand, DLL4, may contribute to NOTCH1-driven leukemias (10). Thus, the majority of T-ALL cases have hyper-activation of the NOTCH signaling pathway. Interestingly, activating mutations affecting *NOTCH1* are also present in 4–13% of B-cell chronic lymphocytic leukemia (B-CLL) cases (11, 12), and very recently frequent non-mutational NOTCH1 activation in B-CLL has also been reported, irrespective of *NOTCH1* mutational status (13). However, differently from T-ALL, the specific role of NOTCH1 signaling in the pathogenesis of B-CLL remains to be established. T-ALL is an aggressive hematological malignancy arising from the malignant transformation and subsequent clonal expansion of immature T-cell precursors. Clinically, T-ALL patients present with diffuse infiltration of the bone marrow (BM) by immature T-cell blasts, high-blood cell counts (hyperleukocytosis) with extramedullary infiltration of lymph nodes and other organs such as the central nervous system (CNS), and the presence of mediastinal masses (14). T-ALL may arise in the BM from thymus settling progenitors endowed with T-lineage potential or thymus resident T-cell precursor cells. These transformed T lymphoblasts under the influence of oncogenic *NOTCH1* activation and collaborating oncogenes spread infiltrating BM cavities and/or thymus with extensive disease already at time of diagnosis. In addition, leukemic cells invade other tissues such as liver, spleen, lymph nodes, and CNS. B-CLL, on the other hand, is a common hematological malignancy characterized by the clonal expansion of non-functional CD5+ B cells in the BM and lymph nodes (15). The putative normal counterparts of this disease, although debated, are considered naïve and memory B cells (16, 17). Interestingly, B-CLL cells in the lymph node are known to harbor frequent NOTCH1 activation independent of mutations (18) and recent findings have shown that NOTCH1 is physiologically expressed and activated in the cells of origin of B-CLL (13). Additionally, approximately 50% of B-CLL cases without *NOTCH1* mutations express the active form of NOTCH1 ICN1 (intracellular portion of NOTCH1), bringing NOTCH1 signaling to the forefront also in this disease.

**Figure 1:**
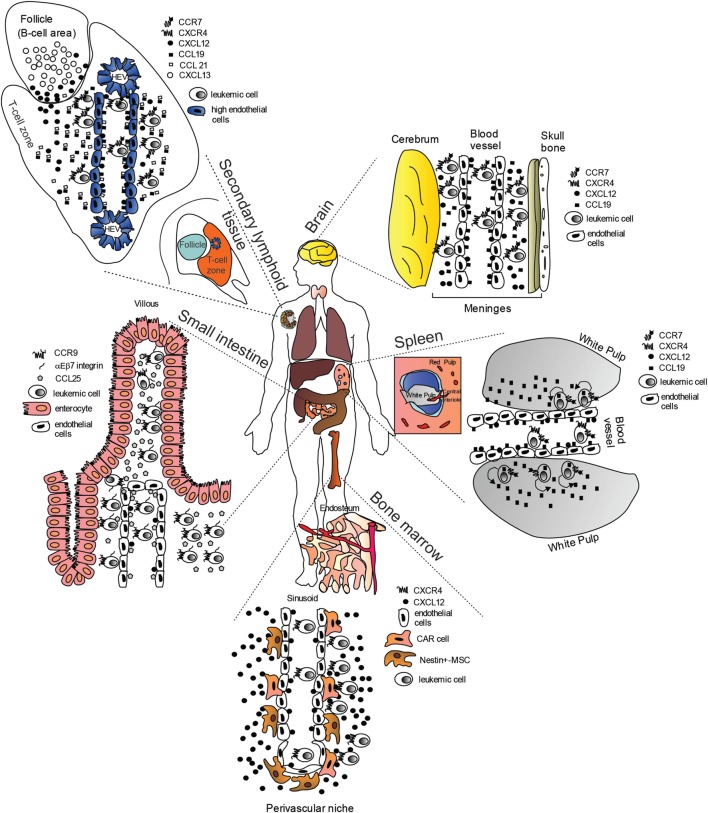
“Cellular highways” hijacked by leukemic cells implicated in T-cell acute lymphoblastic leukemia dissemination (many of the findings may also apply to B-cell chronic lymphocytic leukemia). Under physiological conditions, homeostatic chemokines control cellular migration by directing cells expressing specific chemokine receptors to appropriate locations expressing their cognate chemokine ligands. These cellular highways are also used by leukemic cells. In the brain, CC-chemokine ligand 19 (CCL19) and CXC-chemokine ligand 12 (CXCL12) recruit CC-chemokine receptor 7 (CCR7)- and CXC-chemokine receptor 4 (CXCR4)-expressing leukemic cells from blood vessels. In the spleen, CCL19 recruits CCR7-expressing leukemic cells from blood vessels possibly in combination with CXCL12. Migrated leukemic cells may then activate an autocrine/paracrine secretion of CCL19. CCR7-expressing leukemic cells together with CD62L (not shown) and CXCR4, gain access to secondary lymphoid organs such as lymph nodes (shown) *via* interactions with CCL19, CCL21, peripheral lymph node vascular addressin (not shown) and CXCL12 presented on high-endothelial venules (HEV). Here, leukemic cells are retained, proliferate, and completely substitute the normal tissue architecture. In the bone marrow (BM), CXCR4-expressing leukemic cells are probably initially recruited to the perivascular niche expressing high levels CXCL12, where a leukemic niche is established. Inhibitors of the CXCL12/CXCR4 interaction release leukemic cells from their BM niche, and allow these cells to enter the blood stream. In the small intestine, CCR9-expressing leukemia cells (together with αEβ7 integrin) are recruited by CCL25, where the presence of phosphoinositide-3 kinase-AKT pathway activation contributes to confer a proliferative advantage to leukemic cells in an otherwise non-supportive microenvironment.

Chemokines and their receptors, in particular so-called “homeostatic chemokines” which normally orchestrate leukocyte trafficking and homing during development, have been recently implicated in directing organ-specific metastasis (19, 20). Mechanistic insights on the trafficking of NOTCH-dependent leukemia cells to target organs are still ill-defined, however, recent reports have highlighted the importance of some homing receptors and their ligands (Figure 1) such as: (i) CC-chemokine ligand 19 (CCL19)/CC-chemokine receptor 7 (CCR7) (21); (ii) CXC-chemokine ligand 12 (CXCL12)/CXC-chemokine receptor 4 (CXCR4) (22–24); and (iii) CCL25/CCR9 (25). As leukemic relapse remains a major cause of treatment failure in childhood ALL, with leukemic relapses directly linked to the survival of blasts in the BM and/or distant sites such as CNS, the identification of targetable mechanisms behind this phenomenon are of clear impact.

## Deregulation of NOTCH1 Signaling in Lymphoid Leukemias

*NOTCH* alterations can be found in a broad spectrum of hematological tumors [reviewed in Ref. (26, 27)]. In particular, NOTCH1 and to a lesser extent also *NOTCH2*, resulted the most frequently mutated. NOTCH1 is well known for its role as a master player in the pathogenesis of T-ALL as demonstrated by the high incidence of mutations in this disease (6). Most of these mutations cluster in the negative regulatory region (NRR), which prevents the extracellular receptor from being cleaved by the Disintegrin and metalloproteinase domain-containing protein 10 in the absence of ligand. These mutations mainly include missense substitutions or short insertions or deletions, which lead to receptor destabilization and ligand-independent activation (28). Other mutations in *NOTCH1* truncate the PEST [proline (P), glutamic acid (E), serine (S), threonine (T)-rich protein sequence] domain through non-sense or frameshift events that lead to premature STOP codons in the C-terminal portion of NOTCH1 and increase half-life of ICN1. In addition, in a significant fraction of T-ALL cases, loss of function mutations or deletions in *FBXW7* gene, an ubiquitin ligase implicated in ICN1 turnover, contribute to activation of NOTCH1 signaling in this malignancy (7, 8). Importantly, in about 20% of T-ALL cases, NOTCH1 signaling results strongly activated by the cooperativity of both mechanisms because of dual mutations affecting the NRR and PEST regions of NOTCH1 or the NRR domain together with the *FBXW7* mutations (6–8). The importance of *NOTCH1* mutations has also been extensively validated in murine mouse models of T-ALL. Forced expression of activated forms of Notch1 in murine hematopoietic progenitors determine T-ALL with a penetrance that depends on the strength of oncogenic Notch1 alleles (29, 30). In addition, numerous T-ALL mouse models showed *Notch1* alterations as significant events in T-ALL progression (31, 32). In the context of NOTCH signaling, a role of Notch3 was also established with transgenic mice expressing ICN3 developing T-ALL with high penetrance, demonstrating a potential role for Notch3 in T-ALL (33). In addition, the human T-ALL cell line TALL1, which has wild-type Notch1 but is sensitive to γ-secretase inhibitors (GSI), carries an NRR mutation in *NOTCH3* gene and shows ICN3 overexpression (9, 34). In T-ALL, the oncogenic function of NOTCH1 has been extensively studied and is linked to its capacity to regulate crucial signaling pathways and genes such as nuclear factor-κB (NF-κB), MYC, IGF-1R, and IL-7R all of which contribute to tumor growth and progression (35–39). NOTCH1 also regulates two families of transcriptional repressors Hes and Hey/Hers which in turn exert several downstream effects of NOTCH1 signaling. In particular, Hes1 sustains the phosphoinositide-3 kinase (PI3K)-AKT pathway and NF-κB activation through the direct suppression of phosphatase and tensin homolog (PTEN) and CYLD, respectively (40, 41). Moreover, Hes1 negatively regulates apoptosis of T-ALL cells through the repression of the BBC3/Puma pro-apoptotic factor (42). In addition to the consolidated function of NOTCH1 signaling in promoting anabolic processes and growth, NOTCH1 has been found to regulate some chemokine receptors (CCR5, CCR7, and CCR9; see below) thus orchestrating cell migration in specific microenvironments (21, 43).

As described above, *NOTCH1* mutations have also been described in B-CLL (11, 12). Mutational activation of NOTCH1 has been found in about 8% of B-CLL at diagnosis and at significantly higher frequency during disease progression toward Richter transformation (about 30%), as well as in chemo-refractory B-CLL (about 20%). Differently from T-ALL, *NOTCH1* mutations clustered uniquely in the PEST domain and the 2-bp frameshift deletion (ΔCT7544–7545, P2515fs) is present in about 80% of cases, making it a potential target for screening and specific targeted therapies. Consistent with the association of *NOTCH1* mutations with clinically aggressive forms of the disease, B-CLL with *NOTCH1* mutations at diagnosis have a poor prognosis similar to B-CLL carrying *TP53* disruption and *NOTCH1* mutations and *TP53* disruption tended to distribute in a mutually exclusive pattern (44). The functional role of *NOTCH1* mutations in B-CLL is not completely understood. A recent study showed that ICN1 is expressed in about 50% of peripheral blood B-CLL cases that present wild-type *NOTCH1*, suggesting that alternative mechanisms are involved in NOTCH1 activation in B-CLL (13). Moreover, independent from the mutational status, ICN1^+^ cases expressed a *NOTCH1* gene signature and were sensitive to GSI. Notably, NOTCH1 regulated genes included those with a crucial role in the pathogenesis of B-CLL, including CCND3, BCL2, MCL1, BCR signaling pathway genes, and NF-κB pathway members.

## Chemokines and Chemokine Receptors

Chemokines are small, secreted cytokines with chemotactic properties that are best known for their capacity to mediate immune cell trafficking and lymphoid tissue homeostasis (45, 46). This subfamily of cytokines which comprise over 48 ligands regulate cell trafficking and positioning by activating 20 seven-transmembrane spanning G-protein-coupled chemokine receptors (GPCR). In addition, chemokines can also bind to non-G-protein-coupled seven-transmembrane spanning receptors called atypical chemokine receptors (ACKR), which due to their incapacity to interact with Gi proteins are supposed to act mainly as decoy receptors, scavenging chemokines to help maintain chemokine gradients in tissues. Chemokines are subdivided into four classes based on the position of the first two cysteine (C) residues at their N-terminal protein sequence: CC-chemokines, CXC-chemokines, XC-chemokines, and CX_3_C-chemokines. The chemokine receptor nomenclature is based on the chemokine subclass specificity of the receptor, where L (ligand) is replaced by R (receptor) (47). There is an important degree of promiscuity in the chemokine superfamily, with numerous ligands binding different receptors and vice versa (46). Functionally, chemokines can be divided into “inflammatory” (induced upon inflammation) and “homeostatic” (constitutively expressed in specific tissues or cells) (48). Metaphorically, we can imagine our body as containing “cellular highways” regulated mainly by “homeostatic” chemokines and their receptors through which cells travel to reach specific locations within the body. In this system, chemokines can be envisioned as “traffic directors” responsible for sending cells expressing appropriate chemokine receptors to specific sites. Leukemia cells “hijack” this system to disseminate throughout the body and ensure their survival beyond the primary tumor site (19).

## CXCL12/CXCR4–CXCR7 Signaling

The stromal cell-derived factor-1 (or CXCL12) initially thought to selectively interact with CXCR4, but now known to signal also through CXCR7 or ACKR3 (49), is widely expressed in numerous tissues and cells, including immature osteoblasts and endothelial cells (EC) within the BM, stromal cells in thymus, lungs, liver, brain, and lymph nodes. CXCR4 is also broadly expressed and is frequently overexpressed in cancer (50). Under homeostasis, the CXCL12/CXCR4 axis is crucial for the homing of hematopoietic progenitor cells (HPC) in the BM and their mobilization into the periphery (51). HPC reside in BM “niches” or specialized areas consisting of diverse cells regulating self-renewal, proliferation, and survival of HPC (52). At least two distinct BM niches have been identified, called “osteoblastic/endosteal” and “vascular” niches. In the hypoxic endosteal niche, osteoblasts lining the endosteum are responsible for HPC retention and quiescence maintenance through the intervention of numerous molecules including granulocyte colony-stimulating factor, bone morphogenetic protein, JAG-1/NOTCH1, Angiopoietin-1/Tie2, and osteopontin signaling (53). The vascular niche, localized at the sinusoidal walls, which includes EC, regulates proliferation, differentiation, and mobilization of HPC by secreting stimulatory and inhibitory soluble factors (54). A third niche, formed by CXCL12-abundant reticular cells (CAR), is located in central areas of the BM thus surrounding sinusoidal EC. These CAR cells, which comprise reticular Nestin^+^-mesenchymal stromal cells, as well as leptin receptor positive perivascular stromal cells (55, 56), are essential for the earliest stages of lymphoid development and express high levels of CXCL12, stem cell factor, interleukin-7, Angiopoietin-1, Fms-Related Tyrosine Kinase 3 Ligand, vascular cell adhesion molecule 1, and osteopontin (57–59). These reticular cells promote HPC retention and proliferation.

It is becoming increasingly evident that leukemic cells (and leukemic stem cells) actively interact with the BM microenvironment to promote their proliferation and survival at the expense of normal hematopoiesis (60). Indeed, using a Notch-1-dependent mouse model it has been found that TALL cells suppress normal hematopoiesis through the remodeling of the BM microenvironment by hijacking the proliferative vascular niche and repressing the endosteal/osteoblastic niche (61). The depletion of osteoblasts was due to the aberrant activation of Notch in these cells probably through Hes1-mediated repression of Runx2 transcriptional activity (61). This activation of Notch signaling in osteoblasts (possibly through increased expression of JAG1 or inflammatory cytokines) was associated with a reduced expression of CXCL12 within the stem/perivascular niche. Ultimately, one could envision a feedback loop where leukemic T-cell blasts disrupt homeostatic stem/lymphoid niches leading to compromised hematopoiesis while promoting their own Notch-dependent outgrowth.

T-cell lineage cell production relies on the thymic colonization by BM-exported early progenitors (thymus-seeding progenitors) expressing P-selectin glycoprotein ligand-1 and the chemokine receptors CCR7, CCR9, CXCR4, and possibly CCR5 (62, 63). These cells enter the thymus at the cortico-medullary junction where they undergo T-cell development. In the thymus, CXCL12 seems expressed throughout the cortex (64) by cortical thymic epithelial cells and together with the ligands for CCR7 (CCL21/CCL19) and CCR9 (CCL25) (65) contribute to the gradients required for the step-wise migration of immature thymocytes through the cortex toward the medulla. It has been found that chemokine receptor expression is very dynamic during T-cell development, in fact CCR7 is downregulated during double negative (DN) stages such that pre-positive selection double positive (DP) thymocytes are CCR7^−^, while CD4 and CD8 single positive (SP) thymocytes generated after positive selection re-express CCR7 prior to entering the medulla for tolerance induction (64, 66). On the other hand, DN and DP thymocytes express both CCR9 and CXCR4. The CXCL12/CXCR4 axis seems to have a role beyond acting as a retention signal that maintains DP thymocytes in the cortex, as it critically impacts on the proliferation and survival of DN thymocytes during β-selection acting as a co-stimulator of the pre-T-cell receptor (67). Moreover, CXCL12 may also act as a chemorepellent during the exit of mature SP cells from the thymus into the bloodstream, a process called chemofugetaxis (68). Recently, however, it has been suggested that following positive selection, CXCR4 high CCR9^+^CD69^−^ DP cells downregulate CXCR4 to become CXCR4 low CCR9^+^ CD69^+^ DP cells and subsequently CD4^+^ and CD8^+^ SP cells with very low/undetectable CXCR4 surface expression. Thus, unlike for the DN thymic compartment, CXCR4 expression in DP cells may be dispensable for downstream αβ-T-cell development (64).

CXC-chemokine ligand 12 modulates cancer biology principally through two mechanisms: (i) direct/autocrine effects promoting cancer cell growth, metastasis, and angiogenesis; (ii) by indirect/paracrine effects, including recruitment of CXCR4^+^ cancer cells to CXCL12-expressing organs (BM, liver, thymus, lymph nodes, brain, among others) or CXCR4-expressing stromal cells to tumor sites (69). CXCR4 is overexpressed in many human cancers (70), with numerous studies demonstrating differential expression patterns (nuclear, cytoplasmic, and membrane) which translated in differences in biological behavior of cancers (71). Thus, membrane and/or cytoplasmic CXCR4 promotes tumor cell proliferation and metastasis, while nuclear CXCR4 is ineffective in explicating these functions. The role for CXCL12/CXCR4 axis in the infiltration of extramedullary sites, which commonly express significant levels of CXCL12 is supported by the correlation between high-surface CXCR4 expression by ALL cells (including T-ALL cells) and infiltration of extramedullary organs such as spleen and liver (72).

Recently, Pitt et al. (22) demonstrated that mouse Notch1-dependent T-ALL cells were directly interacting with CXCL12-producing vascular EC, and that this contact was necessary for leukemia maintenance and progression. In addition, murine and human T-ALL cells were shown to express increased cell-surface CXCR4 compared with mature peripheral T cells. Interestingly, this increased expression was not present at the transcript level, suggesting a non-transcriptional mechanism. Indeed, CXCR4 cell-surface expression, results from a balance between endocytosis, intracellular trafficking, and recycling, as well as gene expression (73, 74). CXCR4 internalization requires phosphorylation of its C-terminus, followed by ubiquitination and subsequent β-arrestin-dependent sorting into early endosomes, which are then processed into late endosomes or multivescicular bodies and further fused with lysosomes, ultimately leading to receptor and ligand degradation. The maturation of endosomes entails a cascade controlled by Rab small GTP-ases (75). CXCR4 internalization also depends on a dileucine motif within the C-terminal tail of CXCR4 (76) and numerous proteins including cortactin (77) and PIM1 (73) have been shown to regulate CXCR4 recycling and cell-surface expression. Remarkably, defects in endocytic trafficking of CXCR4 may contribute to increased surface expression and cancer progression (78). In acute myeloid leukemia (AML), a link has been found between PIM1 kinase activity and the surface expression and function of the CXCR4 receptor, with PIM1 expression levels correlating with CXCR4 surface expression (73). Indeed, PIM1 can phosphorylate serine 339 in the C-terminal domain of the CXCR4 receptor (a site critical for receptor recycling) contributing to high-CXCR4 surface expression and function at least in AML and B-CLL (79) cells. Along these lines, it has recently been shown that calcineurin (a serine/threonine protein phosphatase) previously associated with leukemia initiating cell activity (80), affects CXCR4 cell-surface expression at least partially through increased cortactin expression and thus CXCR4 recycling (23). CXCR4 expression was found to be essential for T-ALL maintenance and progression (22, 23) with loss of CXCL12/CXCR4 signaling leading to reduced Myc expression (a transcription factor directly regulated by NOTCH1) and previously linked to leukemia initiating cell activity in T-ALL. Surprisingly, although NOTCH1 has been reported to regulate numerous chemokine receptors in T-ALL (CCR5, CCR7, and CCR9; see below) this is not true for CXCR4 (21, 43), suggesting that NOTCH1 activation is not responsible for the increased CXCR4 expression. Differently in B-CLL cells, which also express high levels of surface CXCR4 and where the CXCL12/CXCR4 axis is regarded as a retention signal in tissue niches, CXCR4 has been shown to be a direct NOTCH1 target (13), suggesting a fundamental role of the NOTCH1-CXCR4 axis in the dissemination of B-CLL cells to lymphoid organs. Some of the main consequences on the biological behavior of T-ALL and B-CLL cells determined by NOTCH1 signaling are summarized in Table 1.

**Table 1:** Functional similarities and differences determined by NOTCH1 in influencing the biological behavior of T-cell acute lymphoblastic leukemia (T-ALL) and B-cell chronic lymphocytic leukemia (B-CLL) cells.

	T-ALL	B-CLL	Reference
Significance of NOTCH1 mutations	Mainly associated with improved therapeutic response and high sensitivity to glucocorticoid therapy	Associated with adverse clinical and biological characteristics (disease progression and chemoresistance)	(44, 81–85)
Effect on CCR7 expression	Transcriptional target (increased expression)	Not known	(21)
Effect on CXCR4 expression	Non-transcriptional increased cell-surface expression^#^	Direct transcriptional target (increased expression^#^)	(13, 22)
Effect on CCR5 expression	Indirect transcriptional target (increased expression)	Generally not expressed	(43)
Effect on CXCR7 expression	Direct transcriptional target (increased expression^#^)	Not known	(86)
Effect on CCR9 expression	Indirect transcriptional target (increased expression)	Generally not expressed	(43)
Effect on c-MYC expression	Direct transcriptional target (increased expression)	Direct transcriptional target (increased expression)	(13, 36, 37)
Main signaling pathways activated to promote cell growth, proliferation, and survival	NF-κB, c-MYC, and PI3K-AKT-mTOR	NF-κB, c-MYC, and MAPK	(13, 35, 87)

*PI3K, phosphatidylinositide 3-kinase; MAPK, mitogen-activated protein kinase; mTOR, mechanistic target of rapamycin; AKT, protein kinase B; NF-κB, nuclear factor kappa B subunit; CCR, CC-chemokine receptor; CXCR, CXC-chemokine receptor; ^#^, to be verified*.

**Figure 2:**
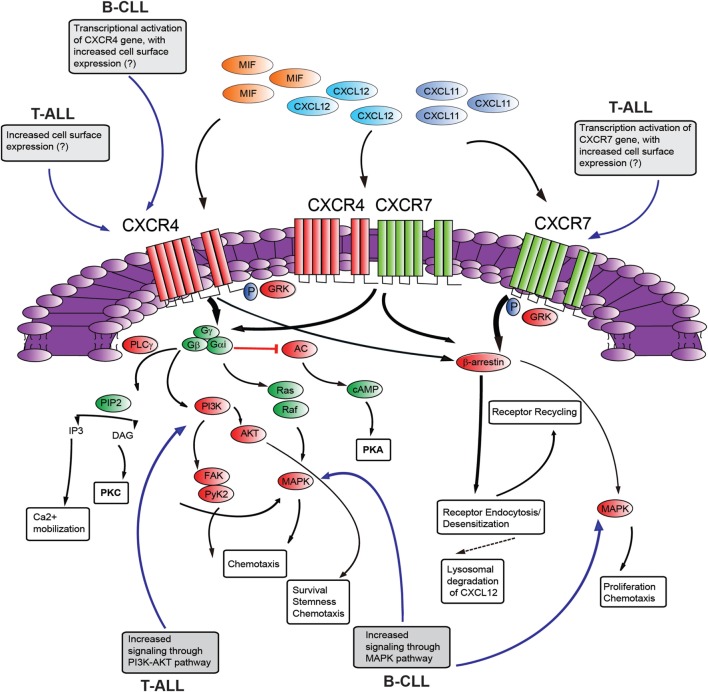
Schematic diagram of putative CXCR4–CXCR7 crosstalk affecting signaling pathways. The influence of NOTCH signaling on the main aspects of this signaling axis is shown in gray boxes [differences between T-cell acute lymphoblastic leukemia (T-ALL) and B-cell chronic lymphocytic leukemia (B-CLL) is presented]. CXCL12 employs two distinct receptors, CXCR4 and CXCR7 which can form homodimers or heterodimers. Additionally, CXCR4 and CXCR7 can act as receptors for macrophage migration inhibitory factor (MIF), while CXCR7 can also bind to CXCL11. Commonly, stimulation of CXCR4 leads to G-protein-coupled chemokine receptors (GPCR) signaling through phosphoinositide-3 kinase (PI3K)/AKT, PLC/IP3, MAPK pathways, and mobilization of Ca2^+^ from intracellular sources. CXCR4/CXCR7 heterodimerization attenuates GPCR signaling, promoting β-arrestin mediated signaling. Activation of CXCR7 triggers β-arrestin mediated signaling. Internalization of the receptors CXCR4 and CXCR7, and subsequent recycling to the cell surface, is also mediated by β-arrestin. Binding of CXCL12 to CXCR7 promotes internalization and scavenging (lysosomal degradation) of CXCL12. AC, adenylyl cyclase; cAMP, cyclic adenosyl monophosphate; PKA, protein kinase A; PLC, phospholipase C; GRK, GPCR kinase; PI3K, phosphatidylinositide 3-kinase; Gα/Gβ/Gγ, heterotrimeric G-protein consisting of subunits α, β, and γ; PIP2, phosphatidylinositol 4,5-bisphosphate; IP3, inositol 1,4,5-bisphosphate; AKT, protein kinase B; MAPK, mitogen-activated protein kinase; FAK, focal adhesion kinase; Pyk-2, proline rich kinase-2; DAG, diacylglycerol; PKC, protein kinase C. “?”, not known; black, pathway activation; red, pathway repression.

CXC-chemokine ligand 12 binding to CXCR4 triggers receptor homo- and heterodimerization, often with CXCR7 (a second chemokine receptor for CXCL12; discussed below), depending on the levels of co-expression (88). The binding of CXCL12 to CXCR4 initiates divergent signaling events that result in numerous responses (possibly cell-type specific) such as chemotaxis, cell survival, and/or proliferation, increase in intracellular calcium and gene transcription (Figure 2). CXCR4 is a GPCR that uses trimeric G-proteins constituted mainly of a Gαi subunit which inhibits adenyl cyclase activity and to a lesser extent a Gαq subunit which activates phospholipase C-β, which leads to inositol 1,4,5 trisphosphate and diacylglycerol production. Ultimately, these events lead to activation of the transcription factor NF-κB, the tyrosine kinase PYK2, Janus kinase-signal transducer and activator of transcription and PI3K-AKT pathways. The βγ dimer instead is mainly involved in Ras activation of ERK1/2 MAPK and activation of PI3K through direct interaction of the βγ dimer with ion channels. Moreover, following ligand-induced CXCR4 phosphorylation by G-protein receptor kinases the interaction with β-arrestin not only mediates clathrin-dependent endocytosis (see above) but also promotes the activation of MAPKs (p38, ERK1/2) and CXCL12-dependent chemotaxis (89). Recently, CXCR7 has been identified as a second receptor for CXCL12, showing a 10-fold higher affinity for this ligand than CXCR4 (49). This receptor is a member of the ACKR subgroup as it does not activate G-proteins after ligand binding (48). This receptor also binds CXCL11 (known ligand of CXCR3) with low affinity and macrophage migration inhibitory factor (90, 91). CXCR7 has been implicated in cell survival and adhesion (92). CXCR7 can act as a scavenger receptor or decoy receptor that removes CXCL12 from the extracellular milieu. Binding of ligands (CXCL12 or CXCL11) to CXCR7 promotes their internalization (49), ligand trafficking to lysosomes (where ligands are degraded), and CXCR7 recycling back to the cell membrane (93). Such CXCR7-dependent regulation of local CXCL12 availability ultimately leads to reduced CXCL12/CXCR4 signaling. On the other hand, the CXCL12 scavenging function of CXCR7 may positively regulate CXCR4-mediated migration by preventing down-regulation of CXCR4 surface expression and function following the exposure to excessive CXCL12 concentrations. In contrast, in cells with primarily intracellular CXCR7 expression and high-CXCR4 surface expression, CXCR7 blockade was not able to alter CXCR4-mediated phosphorylation of ERK and AKT, suggesting that CXCR7 was not necessary for CXCR4 signaling (94). Emerging evidence suggests that CXCR7 is a fully signaling receptor independent of G proteins and can activate intracellular signaling pathways such as AKT, MAPK, Janus kinase-signal transducer, and activator of transcription 3 either by direct modulation, through a β-arrestin-dependent pathway or after heterodimerization with CXCR4 (95). Thus, the relative expression levels of CXCR4 and CXCR7 could critically influence the cellular response to CXCL12. Recently, CXCR7 expression has been found to be very low in normal BM CD34^+^ cells compared with high levels of expression of this receptor in malignant ALL cells and cell lines (96, 97). In addition, particularly high levels of CXCR7 transcript were found in the T-ALL subtype. Analysis of the cellular distribution of CXCR7 in T-ALL cell lines disclosed a rather heterogeneous pattern with a sizable fraction being intracellular in Jurkat cells differently from MOLT4 cells. Interestingly, this different cellular distribution did not modify the functional consequences of CXCR7 silencing, as both cell lines exhibited reduced cell migration in the presence of a CXCL12 gradient (97). Notably, through the use of Notch pathway inhibitors, Asters group has identified a subset of Notch-binding sites in leukemia cell genomes that are dynamic, rapidly changing in occupancy when Notch signaling is modulated (86). These dynamic NOTCH1 sites are highly associated with genes that are directly regulated by Notch and mainly lie in large regulatory switches (termed superenhancers), characterized by exceptionally broad and high levels of H3K27 acetylation (98). The *CXCR7* gene was found to be among these genes with high-dynamic regulatory potential and that are up-regulated following GSI washout in CUTLL1 cells (86). As the list of genes with highly dynamic regulatory potential are enriched for previously identified putative direct NOTCH1 target genes, it will be interesting to validate CXCR7 as a NOTCH1 direct target as this could add a further layer of complexity to the role played by NOTCH1 in promoting T-ALL retention/dissemination.

## CXCL19/CCR7 Signaling

This signaling axis is physiologically important for its role in the development of immune responses, as it normally recruits activated dendritic cells and naïve T cells (expressing CCR7) to draining lymph nodes (expressing high levels of the ligands CCL19/CCL21), thus initiating an adaptive immune response (99). In tumors, CCR7 is often overexpressed and its expression mostly correlates with lymph node metastasis (100). Many leukemia and lymphomas also express CCR7, and this may account for their tropism for lymph nodes (especially T-cell zones) (101). Additionally in B-CLL, the interaction between CXCR5 (expressed at high levels in B-CLL, but not T-ALL cells) and its ligand CXCL13 (produced by resident stromal cells) is responsible for recruiting leukemic cells to lymphoid organs and possibly orchestrates the establishment and maintenance of proliferation centers (pseudofollicles) within these tissues (102). T-ALL patients have increased risk of CNS involvement at diagnosis or relapse, with the mechanisms behind this tropism still ill-defined. Possible entry routes for leukemic cells in the CNS include dissemination to the subarachnoid space from the BM of the skull *via* the bridging veins or from the cerebrospinal fluid *via* the choroid plexus; through brain capillaries to the cerebral parenchyma; infiltration of meninges *via* bony lesions of the skull and possibly traumatic lumbar puncture (24, 103). Buonamici et al. (21) showed that CCR7 signaling regulates CNS infiltration of leukemic T cells, using oncogenic Notch1 mouse models. Indeed, gene expression profiling of uncommitted hematopoietic progenitors expressing oncogenic Notch1 (Notch1-IC) showed significant upregulation of *Ccr7*. NOTCH1-dependent regulation of CCR7 was confirmed in T-ALL cell lines and primary T-ALL samples. Furthermore, overexpression of mouse *ccr7* in a T-ALL cell line not expressing CCR7 (DND41) licenses these cells to specifically infiltrate the brain, possibly through interaction with CCL19 expressed on brain EC. Interestingly, using *ccr6*^−/−^, *ccr7*^−/−^, and *cxcr4*^−/−^ fetal liver progenitors transduced with oncogenic Notch1-IC, *cxcr4* rather than *ccr7* was implicated in CNS infiltration by T-ALL cells, in addition to BM engraftment (24). Significantly, in primary T-ALL samples, high CCR7/CXCR4 mRNA levels correlated with increased risk of CNS involvement (104), although only CCR7 expression had an independent predictive impact on CNS status. Taken together, these data suggest that both CXCR4 and CCR7 play a role in the recruitment of leukemic T cells to the CNS.

The spleen is an important organ involved in extramedullary hematopoiesis and is frequently infiltrated in numerous lymphoid malignancies. There is a high incidence of splenomegaly in ALL, especially T-ALL, with the presence of splenomegaly associated with poorer prognosis of leukemia patients (105). Recent findings from Notch1-dependent leukemia models (106), suggest that the higher levels of CCL19 found in the splenic microenvironment compared with BM could be responsible for the initial homing of these leukemic cells to the spleen (given their expression of CCR7), and at the same time the splenic microenvironment could stimulate the expression of CCL19 by T-ALL cells establishing a positive feed-back loop, leading to further recruitment of leukemic cells to the spleen (106).

## CCL25/CCR9 Signaling

The CCL25/CCR9 chemokine axis normally influences the homing, development, and homeostasis of T cells (107). CCR9 is expressed on the majority of immature DP (CD4^+^CD8^+^) thymocytes, and then is downregulated during their transition to mature SP CD4^+^ or CD8^+^ stage (108). Also, approximately half of all γδ TCR^+^ thymocytes and peripheral γδ-T cells express functional CCR9 (109). The ligand of CCR9, CCL25, is highly expressed not only by cortical and medullary thymic epithelial cells but also by epithelial cells of the small intestine (108). Intriguingly, a case report of a pediatric T-ALL expressing CCR9 (and CD103 or αEβ7 integrin) at diagnosis, that switched to acute myeloid leukemia at relapse with disease localization to the gut has been reported (110), suggesting a role for CCR9 in the gut tropism of these leukemic cells. Recently, an elegant study found that conditional postnatal knockdown of *Pten* (*shPten*) in the hematopoietic compartment produced a highly disseminated T-ALL with the majority of leukemias harboring activating mutations in the Notch1 PEST domain (25). These *shPten* leukemias expressed high levels of CCR9 and showed marked dissemination to the intestine (and liver). Surprisingly, PTEN reactivation had no effect on tumor growth in the lymph nodes or spleen, while it markedly decreased tumor infiltration into intestine and liver, suggesting that the impact of Pten expression on disease progression is dictated by the anatomical site of leukemic disease. Subsequent experiments to determine how PTEN influences T-ALL homing and survival in the intestine disclosed that reduced PTEN expression (through *Pten* knockdown) sensitized leukemia cells to CCL25-induced Akt phosphorylation leading to their increased migration in transwell assays, and this effect was largely abrogated following PTEN re-expression (25). These findings suggest that leukemic cells with PTEN suppression or loss are facilitated in dissemination to distant sites such as the intestine (if they express CCR9) and amplify weak environmental cues (such as CCL25 signaling) that enable their survival. Consistent with this notion, stimulation of T-ALL cells with CCL25 has been reported to enhance their resistance to TNF-α mediated apoptosis (through the induction of the inhibitor of apoptosis protein Livin) partly through the activation of c-*jun*-NH2-kinase 1 (111). Interestingly, the Notch pathway has been shown to indirectly control the expression levels of CCR9 (and CCR5) in T-ALL cell lines and patient-derived primary leukemia cells, and subsequent biological effects such as cell proliferation and migration (43). It could thus be speculated that PTEN suppression together with NOTCH1 activation (frequently observed in human T-ALL) could cooperate to enhance migration to specific anatomical sites such as the intestine (through the increased expression of selected chemokine receptors such as CCR9) and confer a proliferative advantage in an otherwise non-supportive microenvironment (CCL25-expressing sites).

## Conclusion and Perspectives

ALL is the most common malignancy in children, with 15% showing markers for the T-lineage (T-ALL). Of these, approximately 20% still die due to disease relapse. Instead in adults, T-ALL represents around 25% of ALL cases, with approximately 50% dying due to disease relapse notwithstanding current combination chemotherapy (112, 113). B-CLL is the most common human leukemia in adults, with patients often presenting an indolent course, surviving for a number of years with relatively mild symptoms (15). In ALL, leukemia relapses have been directly linked to the survival of blasts in organs such as CNS or testes in addition to BM (103). Infiltration of distant organs such as CNS is frequently observed in T-ALL and is an important obstacle for long-term remission. Many genes are implicated in the pathogenesis of T-ALL, including NOTCH, with *NOTCH1* mutations being identified in over half of T-ALL patients (6). Although the mechanisms of normal T-cell homing to lymphoid organs and trans-endothelial migration are relatively well known, the mechanisms exploited by leukemic T cells to gain access to target organs remain elusive. Homeostatic chemokines are considered pivotal molecules in promoting metastasis in solid tumors (19), and may help to account for the non-random metastatic destinations encountered in different neoplasia. In B-CLL, NOTCH1 activation probably reflects the constitutive, dysregulated expression of a physiological signal (13). *NOTCH1* mutations in T-ALL hijack the physiological role of NOTCH signaling during thymocyte development (114) with oncogenic *NOTCH1* alterations expressed in HPC often used as models of human T-ALL to gain mechanistic insights. Mainly through the use of these NOTCH1-dependent leukemias it is emerging that homeostatic chemokines and their receptors are critically involved not only in dictating medullary and extramedullary dissemination but also directly affecting the viability and growth of nascent leukemic niches. Recent studies showing that surface chemokine receptor expression and function may not correlate with mRNA transcript levels and that defects in recycling or endocytic trafficking of chemokine receptors may contribute to cancer progression add a new layer of complexity to the mechanisms acting to fine-tune the functional consequences of chemokine signaling. Thus, future studies evaluating the significance of chemokine receptor expression/signaling will need to go beyond mRNA expression levels, but will also have to take into account receptor phosphorylation, ubiquitination, recycling, and internalization rates. In particular, it may also be worth revisiting the role of CXCL12 biology in T-ALL (and possibly B-CLL) from the CXCR7 perspective. Intriguing are also recent observations that anti-tumor therapies (radiation and chemotherapy, among others) promote a hypoxic environment (19), which through the stabilization of hypoxia-inducible factors can increase the expression of chemokine receptors such as CXCR4 (115); conversely, other chemotherapies can downregulate chemokine receptor expression (19). Thus, some current therapies aimed at killing tumor cells may actually promote a more aggressive phenotype in the surviving cells (116). In B-CLL, the Bruton’s tyrosine kinase inhibitor, Ibrutinib, has been shown to determine early lymphocytosis and organomegaly reduction followed by normal cell count restoration, possibly in part due to its effects on CXCR4 expression (117). The effects of contemporary chemotherapy regimens used in T-ALL on chemokine receptor expression remain to be elucidated. Comprehensively, although numerous studies have focused on the role of chemokine receptors and their regulation by NOTCH, much less is known on downstream signals such as integrin activation or actin remodeling dynamics.

Currently, clinically approved targeted therapies to impede organ infiltration in acute leukemia are lacking. Of the chemokine axes that can be targeted, the CXCL12/CXCR4–CXCR7 axis seems most promising in T-ALL, as monotherapy with the selective CXCR4 antagonist, AMD3465, was highly effective in suppressing human disease in a xenograft model (22). However, monotherapy with CXCR4 inhibitors in other malignancies has more modest anti-leukemic effects (including B-CLL), as it mainly sensitized leukemic cells to conventional or targeted therapies through the mobilization of the leukemic cells into the periphery (102, 118, 119). Thus, it is likely that combination therapies comprising chemokine receptors antagonists together with conventional chemotherapeutic agents or specific targeted therapies such as NOTCH1 inhibitors will be required to eradicate the disease and prevent relapse.

## Author Contributions

All authors listed have made a substantial, direct, and intellectual contribution to the work and approved it for publication.

## Conflict of Interest Statement

The authors declare that the research was conducted in the absence of any commercial or financial relationships that could be construed as a potential conflict of interest.
